# Atrial Cardiopathy and Cryptogenic Stroke

**DOI:** 10.3389/fneur.2022.839398

**Published:** 2022-02-22

**Authors:** Yuji Kato, Shinichi Takahashi

**Affiliations:** Department of Neurology and Cerebrovascular Medicine, Saitama Medical University International Medical Center, Hidaka, Japan

**Keywords:** atrial cardiopathy, atrial dysfunction, atrial fibrillation, cardioembolic stroke subtype, embolic stroke of undetermined source

## Abstract

Recent advances in pathophysiology suggest that a pathological atrial substrate can cause embolic stroke even in patients without atrial fibrillation (AF). This pathological condition is called “atrial cardiopathy”, which indicates atrial structural and functional disorders that can precede AF. The objective of this narrative review was to provide a current overview of atrial cardiopathy and cryptogenic stroke. We searched the PubMed database and summarized the recent findings of the identified studies, including the pathogenesis of atrial cardiopathy, biomarkers of atrial cardiopathy, relationship between atrial cardiopathy and cryptogenic stroke, and therapeutic interventions for atrial cardiopathy. Abnormal atrial substrate (atrial cardiopathy) that leads to AF can result in embolic stroke before developing AF, and may explain the source of cryptogenic stroke in some patients. Although there are several potential biomarkers indicative of atrial cardiopathy, P-wave terminal force in lead V1 (>5,000 μV^*^ ms), N-terminal pro-brain natriuretic peptide (>250 pg/ml), and left atrial enlargement are currently promising biomarkers for the diagnosis of atrial cardiopathy. Because the optimal combination and thresholds of biomarkers for diagnosing atrial cardiopathy remain uncertain, atrial cardiopathy represents a spectrum disorder. The concept of atrial cardiopathy appears to be most valuable as a starting point for therapeutic intervention to prevent stroke. Validation of the diagnosis of atrial cardiopathy and whether it can be used as a new therapeutic target for direct oral anticoagulants are currently being covered in the ARCADIA trial.

## Introduction

Approximately one fourth of all ischemic stroke patients are classified as cryptogenic strokes, most of which are caused by an embolic mechanism (1). Cryptogenic strokes have usually meant a non-lacunar infarction without proximal arterial stenosis or cardioembolic sources; however, there is neither a widely accepted definition nor a required diagnostic assessment. This has inhibited clinical research into optimal preventive therapy for cryptogenic strokes. In 2014, the Cryptogenic Stroke/ESUS International Working Group proposed the classification of a new subgroup of cryptogenic stroke: embolic stroke of undetermined source (ESUS) (1). The definition of ESUS is as follows: (1) detection of a non-lacunar infarct on brain computed tomography/magnetic resonance imaging; (2) exclusion of ≥50% atherosclerotic stenosis proximal to the infarct with any imaging modality (catheter, magnetic resonance, computed tomography angiography, or ultrasonography); (3) exclusion of a major-risk cardioembolic source with echocardiography and cardiac monitoring for ≥24 h; and (4) no other specific causes (e.g., arteritis, dissection, migraine, and drug misuse) (1). The concept of ESUS has contributed to facilitating clinical trials testing direct-acting oral anticoagulants (DOAC) for the secondary prevention of ESUS.

Although ESUS represents a heterogeneous clinical entity, the hypothesis that covert paroxysmal AF is the primary cause of ESUS is widely affirmed and has contributed to the current practice of performing long-term cardiac rhythm monitoring after ESUS. However, an implantable cardiac monitor detected AF in only 30% of patients during a 3-year period (2). This implies that AF may not be a necessary condition for cardioembolism.

Recent advances in pathophysiology have suggested that left atrial degeneration, including chamber dilation, remodeling, fibrosis, and damage to endothelial cells and cardiomyocytes, can induce thrombus generation and cause embolism, even in patients without AF (3, 4). This condition was defined as atrial cardiopathy, a term used to describe atrial structural and functional disorders that can precede AF (5). Atrial cardiopathy increases the stroke risk in patients with AF and is likely to also increase the stroke risk in patients without AF. In this article, we review the pathogenesis of atrial cardiopathy, its biomarkers, its association with stroke, and the potential for therapeutic intervention.

## Pathogenesis Of Atrial Cardiopathy

The mechanism of thrombosis has long been recognized as Virchow's triad, summarized as stasis, hypercoagulability, and endothelial damage. In the case of AF, thrombus formation is induced by decreased left atrial appendage (LAA) flow velocity, activation of the coagulation cascade, and left atrial enlargement (LAE) and fibrosis. Recent findings suggest that the causal association between AF and stroke is not simple and still not fully understood.

**Figure 1:**
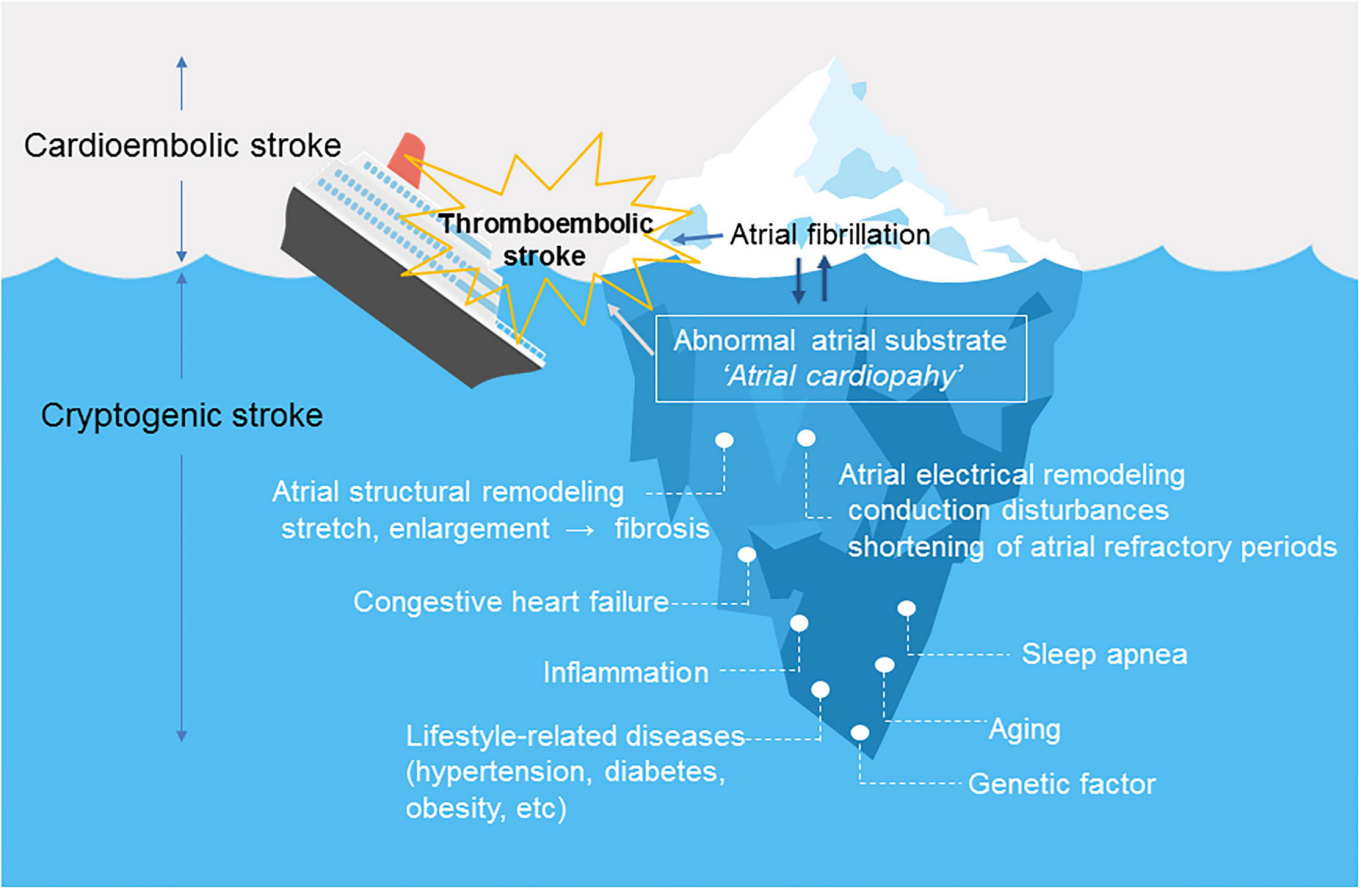
Atrial cardiopathy as a cause of thromboembolic stroke. Atrial fibrillation is likely only a marker (tip of the iceberg) for underlying abnormal atrial substrate or “atrial cardiopathy”. Underlying atrial cardiopathy can cause stroke, even in patients without atrial fibrillation. Once atrial fibrillation develops, the dysrhythmia causes structural and electrical remodeling, which further increases the risk of thromboembolism.

Figure 1 shows the relationship between atrial cardiopathy and thromboembolic stroke. Aging and lifestyle-related diseases promote atrial damage, including stretching and enlargement of the left atrium. Over time, this causes increased fibrosis of the left atrial myocardium under a process modulated by genetic predisposition. The resulting fibrotic changes promote left atrial remodeling, including structural and electrical changes (6), which forms abnormal atrial substrate (atrial cardiopathy) that can lead to the occurrence of AF and embolic stroke (3, 7). AF leads to further atrial remodeling. It is important to note that atrial cardiopathy can predispose to embolic stroke even in patients without AF.

## Biomarkers Of Atrial Cardiopathy

There are still no established criteria for the diagnosis of atrial cardiopathy. However, researchers have attempted to identify atrial cardiopathy biomarkers associated with stroke risk factors found in ESUS patients. Table 1 shows the potential biomarkers of atrial cardiopathy associated with the risk of stroke. These biomarkers are classified as electrophysiological, structural, hemodynamic, serological, and genetic markers.

**Table 1:** Potential biomarkers associated with atrial cardiopathy and stroke risk.

**Biomarkers classified into categories**	**Specific examples**
**Electrophysiological**	
Atrial fibrillation	Subclinical atrial fibrillation
*P*-wave morphology	*P*-wave terminal force in lead V1
Paroxysmal supraventricular tachycardia	
Atrial ectopy	Excessive supraventricular ectopic activity
**Structural**	
Left atrial size	Left atrial enlargement, left atrial volume index
LAA morphology	Non-chicken wing type
Myocardial fibrosis	Regions of delayed gadolinium enhancement on cardiac MRI
**Hemodynamic**	
LAA flow velocity	Low flow velocity
**Serological**	
NT-proBNP, BNP	
Hs-cTnT	
**Genetic**	
Polymorphisms	rs2200733, rs10033464

*LAA, left atrial appendage; MRI, magnetic resonance imaging; NT-proBNP, N-terminal pro-brain natriuretic peptide; BNP: brain natriuretic peptide; Hs-cTnT, high-sensitivity cardiac troponin T*.

## Electrophysiological Markers

Symptomatic stroke may be an initial clinical manifestation of underlying AF. Two randomized trials have found that prolonged rhythm monitoring of outpatients after ESUS results in a higher detection rate of AF than with standard monitoring (2, 8). Although even brief subclinical episodes of AF are associated with the occurrence of stroke (9), the causal association between ischemic stroke and AF remains circumstantial (3). Both the ASSERT and TRENDS studies reported that in only 8–28% of patients, subclinical AF was detected within 30 days before stroke or systemic embolism (10, 11). The absence of temporality implies that AF itself may not be the direct cause of stroke in patients with short-term AF. Instead, AF may be a risk indicator for embolic stroke associated with underlying atrial dysfunction.

**Figure 2:**
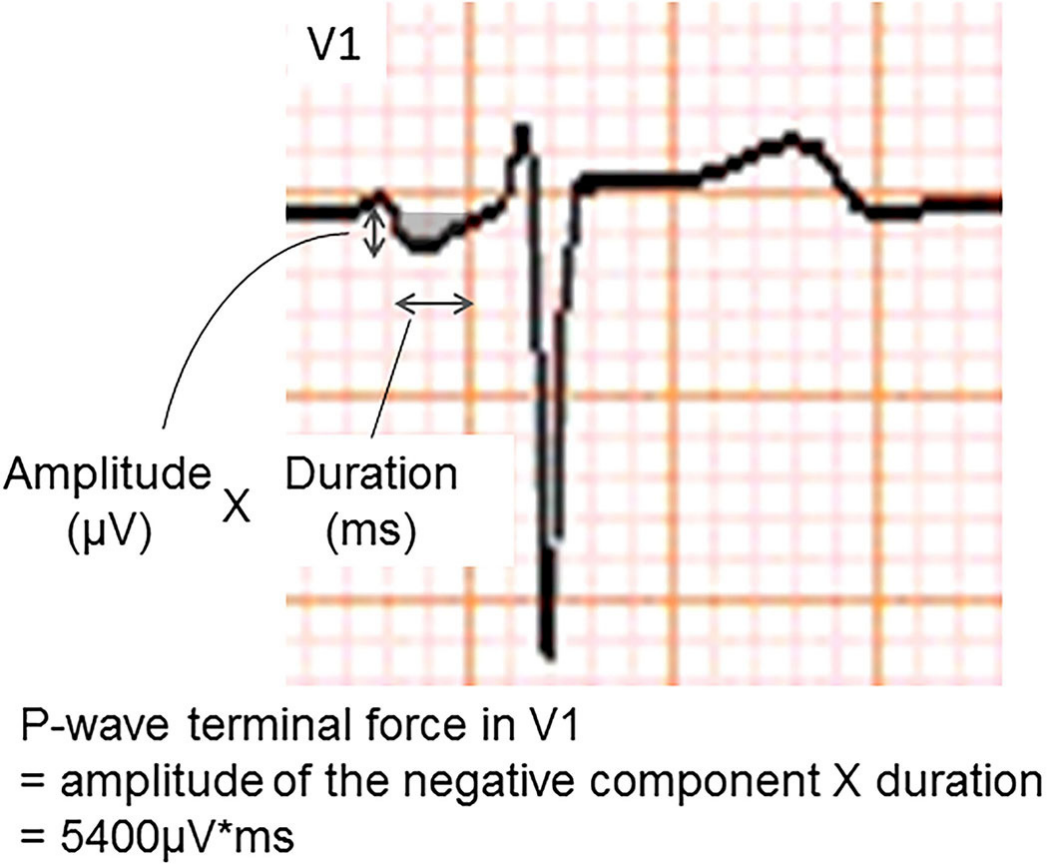
An example of abnormal *P*-wave terminal force in lead V1. The *P*-wave terminal force in lead V1 was calculated as multiplying the amplitude and duration of the terminal negative part of the *P*-wave in lead V1.

**Figure 3:**
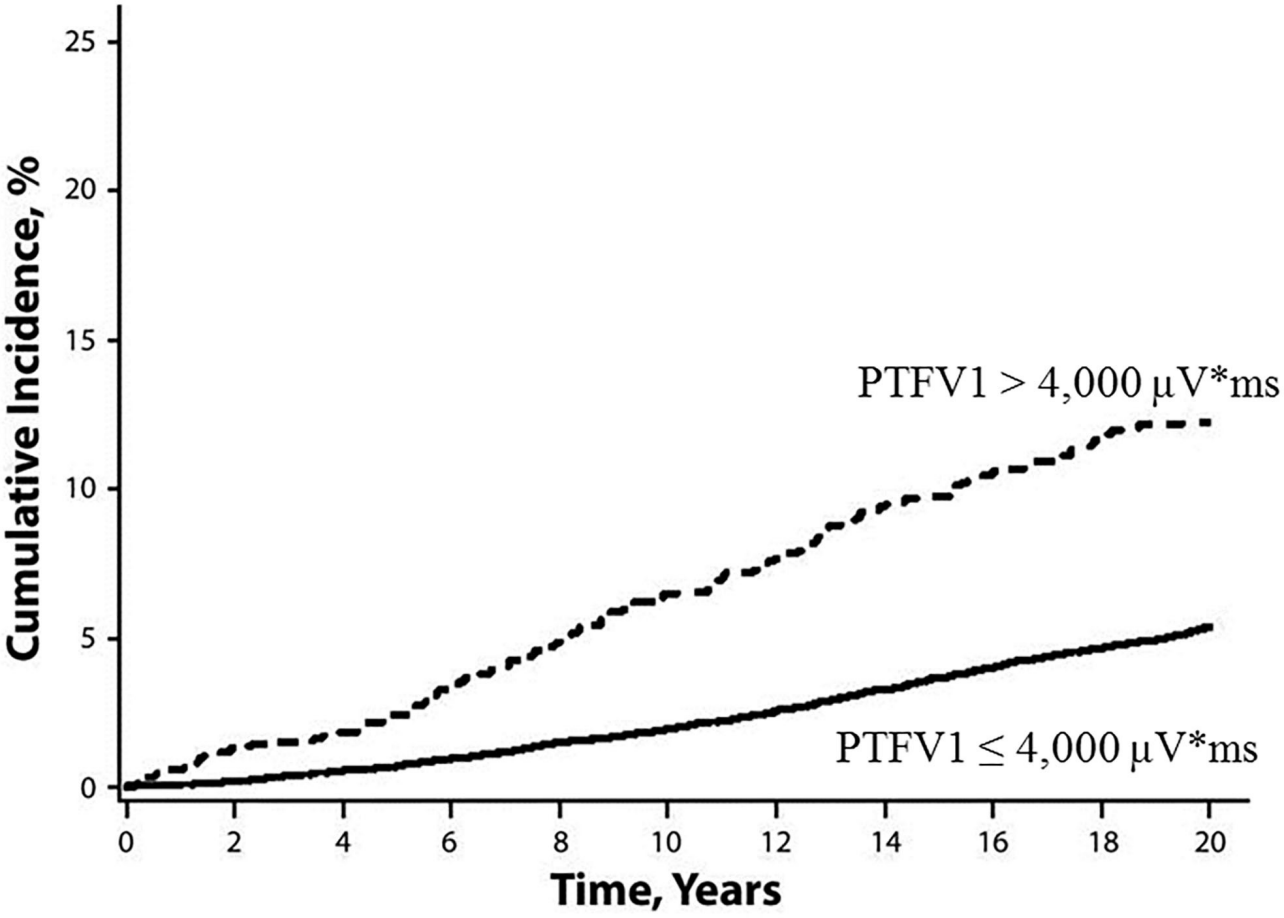
Cumulative incidence of ischemic stroke, stratified by baseline PTFV1 (15). Ischemic stroke occurred more frequently in those with abnormal PTFV1 (>4,000 μV* ms) than in those without (log-rank test *p* < 0.001). PTFV1, *P*-wave terminal force in lead V1.

The *P*-wave terminal force in lead V1 (PTFV1) (Figure 2) becomes prolonged with left atrial hypertrophy, fibrosis, and increased filling pressure, and is thought to reflect structural and functional impairment of the left atrium (12, 13). In the Multi-Ethnic Study of Atherosclerosis, which included 6,741 participants aged 45–85 years without a history of cardiovascular disease, stroke, or AF, PTFV1 was associated with a higher incidence of ischemic stroke [hazard ratio (HR) per standard deviation 1.21, 95% confidence interval (CI) 1.02–1.44] than AF (HR per standard deviation 1.11, 95% CI 1.03–1.21) after a mean of 8.5 years of observation (14). In the Atherosclerosis Risk in Communities study of 14,542 AF-free participants observed for a median of 22 years, ischemic stroke occurred more frequently in those with abnormal PTFV1 (>4,000 μV^*^ ms) than in those without (6.3 vs. 2.9 per 1,000 person-years, respectively, *p* < 0.001; Figure 3). Abnormal PTFV1 was associated with ischemic stroke (HR 1.33, 95% CI 1.11–1.59) and non-lacunar infarction (HR 1.49, 95% CI 1.07–2.07) but not with lacunar infarction (HR 0.89, 95% CI 0.57–1.40) (15).

Paroxysmal supraventricular tachycardia (PSVT) is a benign rhythm disorder that has been associated with palpitations but that does not increase the risk of stroke. However, a follow-up of 169 patients with PSVT revealed that 12% developed AF within 1 year and 19% developed AF within a mean of 31 months (16). In addition, a large cohort study conducted in California found that PSVT was associated with ischemic stroke even in the absence of AF (adjusted HR 2.10, 95% CI 1.69–2.62) (17).

**Figure 4:**
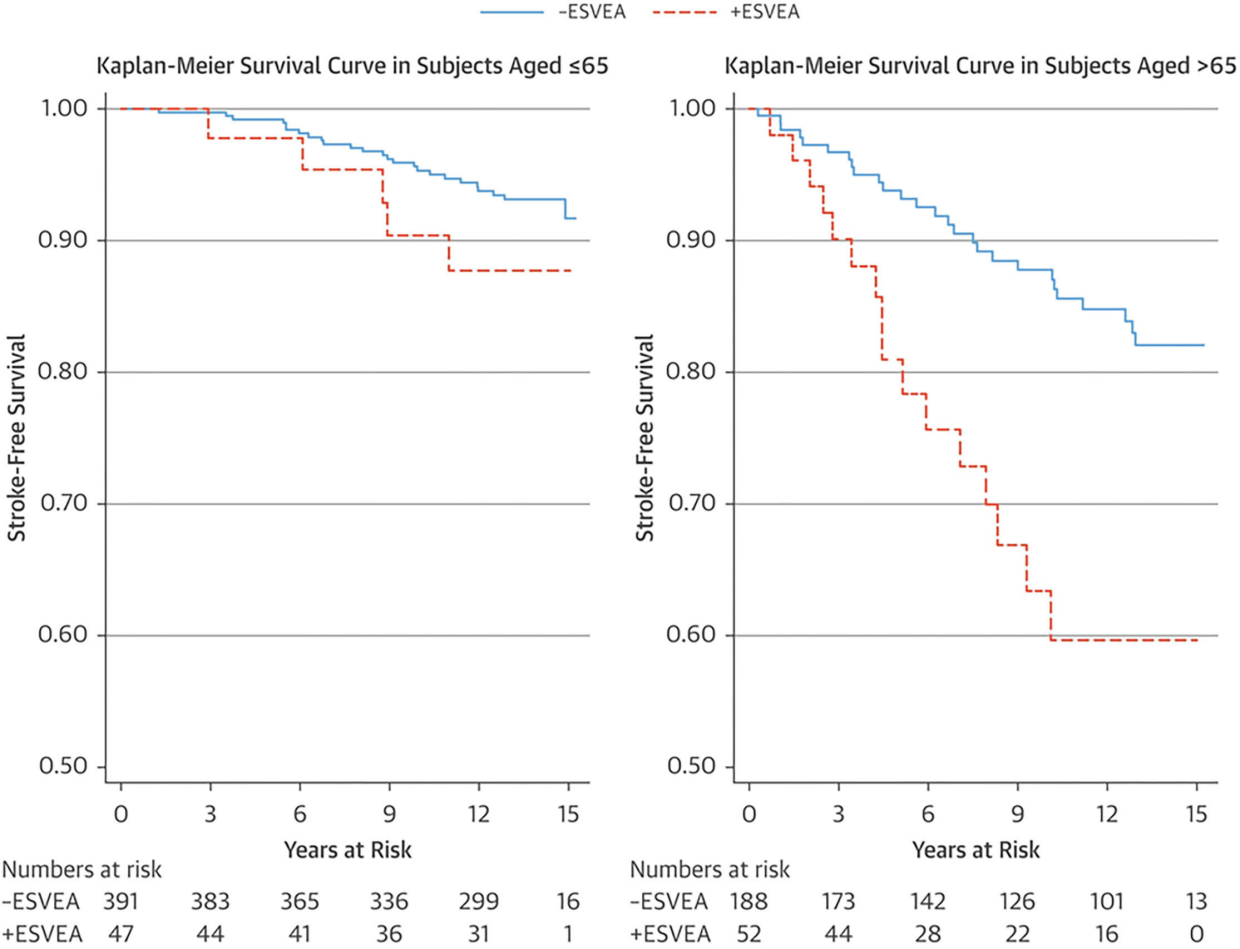
Kaplan–Meier survival estimate of stroke-free survival stratified by age group and ESVEA (18). The incidence of stroke associated with ESVEA was higher in patients >65 years of age (*p* = 0.0007), but not in patients ≤ 65 years of age (*p* = 0.2086). ESVEA, excessive supraventricular ectopic activity.

An ectopic atrial rhythm is also considered a risk factor for ischemic stroke. In the Copenhagen Holter Study, 678 patients aged 55–75 years without a history of cardiovascular disease, stroke, or AF underwent 48 hours of mobile electrocardiography (ECG) and were followed-up for 15 years (18). Excessive supraventricular ectopic activity (ESVEA) was defined as the presence of either ≥30 premature atrial contractions/hour or any runs that lasted for ≥1 h. Ninety-nine patients (15%) had ESVEA during follow-up. The incidence of stroke was higher in patients with ESVEA than those without in >65 years of age (38.5 vs. 13.6 per 1,000 person-years, respectively, *p* = 0.0007), but not in ≤ 65 years of age (8.9 vs. 5.0 per 1,000 person-years, respectively, *p* = 0.2086) (Figure 4). After adjusting for risk factors and censoring for the development of AF, the risk of stroke in patients with ESVEA was nearly twice as great as in those without ESVEA (HR 1.96, 95% CI 1.10–3.49). Of the patients with ESVEA who had cerebral infarction, 14.3% developed AF before their stroke. The incidence of cerebral infarction in patients with ESVEA and a CHA2DS2-VASc score of ≥2 points was 2.4%/year, indicating a risk similar to that of AF.

## Structural Markers

Structural abnormality of the left atrium indicates the need to search for findings of atrial cardiopathy. Population-based studies have shown that LAE is associated with developing AF (19) and incident ischemic stroke after multivariable adjustment including AF. The Framingham study reported that LAE is a significant predictor of stroke in men (adjusted HR per 10 mm increase 2.4, 95% CI 1.6–3.7) and women (adjusted HR per 10 mm increase 1.4, 95% CI 0.9–2.1) (20). In the Northern Manhattan Stroke Study of 655 patients with previous ischemic stroke, moderate to severe LAE (≥47 mm in men and ≥43 mm in women) was significantly associated with recurrent cardioembolic/cryptogenic stroke compared with normal LAE (adjusted HR 2.83, 95% 1.03–7.81) (21). However, one limitation of these studies is that they used left atrial diameter, which does not fully represent the true three-dimensional size of the left atrium. It has recently been shown that the left atrial volume index to the subject's body surface area is a superior indicator of left atrial size in terms of predicting cardiovascular outcomes (22). Furthermore, a recent study revealed that the left atrial volume index to the subject's body surface area is independently associated with the development of AF in ESUS patients (adjusted odds ratio per mL/m^2^ 1.09, 95% CI 1.02–1.15, *p* = 0.007) (23).

The fundamental cause of AF is thought to be atrial fibrosis (24, 25). On cardiac magnetic resonance imaging, fibrotic areas are visualized as areas of delayed gadolinium enhancement (26). The presence of left atrial fibrosis on cardiac magnetic resonance imaging appears to correlate with stroke risk. A cross-sectional analysis of 387 AF patients revealed that patients with previous strokes had significantly higher levels of fibrosis than those who had not experienced a stroke (27). In a multivariate analysis, the presence of fibrosis was a better predictor of stroke risk than the CHADS2 score. A recent study revealed that the prevalence of left atrial fibrosis in ESUS patients was higher than that in patients with stroke due to other causes (*p* = 0.03) and similar to that in stroke patients with AF (*p* = 0.22) (28).

The risk of stroke may also be associated with the morphological features of the LAA (29). In a previous study of AF patients, a non-chicken wing LAA morphology was more likely to be associated with ischemic stroke (30). In a retrospective study of 172 stroke patients, the prevalence of non-chicken wing LAA morphology on chest computed tomography tended to be higher in patients with cardioembolic stroke (58.7%) and ESUS (58.8%) than in those with noncardioembolic stroke (46.3%); however, the difference did not achieve statistical significance (31).

## Hemodynamic Markers

The LAA appears to play an important role in intracardiac thrombus generation in AF patients. More than 90% of intracardiac thrombi in AF patients are identified in the LAA (32), and a decreased LAA flow velocity is thought to be associated with the generation of stasis (33). Among 721 patients who underwent transesophageal echocardiography in a *post hoc* analysis of the Stroke Prevention in Atrial Fibrillation-III trial, reduced LAA flow velocity (<20 cm/s) was associated with thrombus formation and subsequent cardioembolic stroke (33). Similarly, a cross-sectional study of 909 stroke patients with or without AF found that decreased LAA flow velocity (<60 cm/s) was associated with clinically elevated stroke severity (34), and an observational study of 786 patients with cryptogenic stroke found that decreased LAA flow velocity on transesophageal echocardiography was associated with multiple infarcts (35). These results indicate that decreased flow velocity in the LAA is an additional risk factor for cardioembolic stroke, regardless of whether AF exists.

## Serological Markers

Serum biomarkers could be informative to detect a high risk of paroxysmal AF in patients with stroke and to preselect patients who require long-term cardiac monitoring after stroke. For example, B-type natriuretic peptide (BNP) and N-terminal pro-brain natriuretic peptide (NT-proBNP) are released by the cardiac myocytes in response to stretch and are therefore increased in patients with heart failure, AF, and ventricular strain. Thus, BNP and NT-proBNP levels have been proposed as indicators of cardioembolic origin in stroke of unknown cause (36).

In a case-cohort analysis of the Reasons for Geographic and Racial Differences in Stroke cohort (*n* = 1,502, mean follow-up 5.4 years), patients in the highest quartile of serum NT-proBNP concentration had a 3-fold higher stroke risk than those in the lowest quartile (HR 2.9, 95% CI 1.9–4.5); the association was strongest for cardioembolic subtypes (HR 9.1, 95% CI 2.9–29.2), suggesting that the stroke risk was due to embolism (37). A subanalysis of the Find-AF_RANDOMISED_ trial of 398 stroke patients without AF showed that the median BNP level was higher in patients with paroxysmal AF detected by frequent and longer Holter ECG monitoring than in patients without AF (57.8 vs. 28.3 pg/mL, respectively, *p* = 0.0003) (38). A BNP cutoff value of ≥100 pg/mL was useful to preselect stroke patients who required frequent and longer Holter ECG monitoring.

Cardiac troponin (cTnT) is a biomarker of myocardial damage often used to detect myocardial ischemia. Highly sensitive assays can determine cTnT concentrations of less than one-tenth that detected in conventional assays used for the identification of acute myocardial ischemia. In the ARIC study of 10,902 stroke-free patients observed for a mean of 11.3 years, a highly sensitive assay concentration of cTnT in the highest quintile was significantly associated with cardioembolic stroke (HR 2.63, 95% CI 1.28–5.37, *p* = 0.003) compared with the lowest quintile, but not with lacunar infarction (39).

## Genetic Markers

Recently, a genome-wide association study found a haplotype block on chromosome 4q25 associated with AF (40). In addition, two single nucleotide polymorphisms (rs2200733 and rs10033464) have been reported to be associated with the development of AF (41) and ischemic stroke, especially the cardioembolic subtype, even in patients in whom active AF has not been detected (42). While paroxysmal AF may be underdiagnosed in these patients, their genetic predisposition may involve potential left atrial abnormalities that lead independently to both AF and stroke.

## Atrial Cardiopathy And Cryptogenic Stroke

Although specific diagnostic criteria for atrial cardiopathy and the thresholds indicative of increased stroke risk are still being developed, it appears that atrial cardiopathy can be provisionally diagnosed by the presence of one or a few of the biomarkers of atrial dysfunction discussed above. In the Cardiovascular Health Study, among 3,723 participants without stroke and AF at baseline, 585 participants developed an incident ischemic stroke during a median 12.9 years of follow-up (43). PTFV1 (HR per 1,000 μV^*^ ms 1.04, 95% CI 1.001–1.08), NT-proBNP (HR per doubling of NT-proBNP 1.09, 95% CI 1.03–1.16), and incident AF (HR 2.04, 95% CI 1.67–2.48) were each independently associated with incident ischemic stroke, but not the left atrial diameter (>4.3 cm in women, >4.7 cm in men).

In a cross-sectional study of 846 stroke patients, the prevalence of atrial cardiopathy (defined as PTFV1 >5,000 μV^*^ ms or severe LAE) was higher in ESUS patients than in patients with noncardioembolic stroke (26.6 vs. 12.1%, respectively, in large artery atherosclerosis vs. 16.9% in small artery disease; *p* = 0.001) (44).

In a subanalysis of 3,983 eligible patients from the New Approach Rivaroxaban Inhibition of Factor Xa in a Global Trial vs. ASA to Prevent Embolism in Embolic Stroke of Undetermined Source (NAVIGATE ESUS), 235 (5.9%) patients had LAE, 939 (23.6%) had ipsilateral carotid plaque to ischemic stroke, and 94 (2.4%) had both (45). Although the common risk factors were male sex, Caucasian ethnicity, hypertension, tobacco use, and coronary artery disease, increasing atrial diameter was not associated with carotid plaque after adjustment (odds ratio per cm, 1.1, 95% CI 1.0–1.2, *p* = 0.08). There was also no association between line of atrial cardiopathy (premature atrial contractions on Holter ECG, new onset of AF) and carotid plaque formation. These results suggest that atrial cardiopathy and carotid plaque are likely separate and nonoverlapping risk factors in patients with ESUS.

## Therapeutic Interventions For Atrial Cardiopathy

Current knowledge suggests that atrial cardiopathy is likely to be the stroke etiologic subtype most similar to AF and may benefit from anticoagulation, particularly DOAC therapy. In fact, secondary analyses of the NAVIGATE ESUS trial found that rivaroxaban was superior to aspirin in the subset of patients with LAE (>4.6 cm) (HR 0.26, 95% CI 0.07–0.94, *p* = 0.02) (46), but not in the subset of patients with ipsilateral nonstenosing plaque (47).

The AtRial Cardiopathy and Antithrombotic Drugs In prevention After cryptogenic stroke (ARCADIA) trial is currently underway (48), with the primary objective to validate the hypothesis that a DOAC (apixaban) is more effective than aspirin for stroke prevention in cryptogenic stroke patients with atrial cardiopathy. In the ARCADIA trial, atrial cardiopathy was defined as the presence of one or more of the following: PTFV1 >5,000 μV^*^ ms, NT-proBNP >250 pg/ml, and left atrial diameter index ≥ 3 cm/m^2^.

## Conclusion

Atrial cardiopathy should be considered one of the mechanisms of ESUS. Although there are several potential biomarkers indicative of atrial cardiopathy, PTFV1 (>5,000 μV^*^ ms), NT-proBNP (>250 pg/ml), and left atrial enlargement are currently promising biomarkers for the diagnosis of atrial cardiopathy. Because the best combination and thresholds of biomarkers for diagnosing atrial cardiopathy remain uncertain, atrial cardiopathy represents a spectrum disorder. The concept of atrial cardiopathy is useful as a starting point for therapeutic intervention to prevent stroke. The ARCADIA trial is currently being performed to validate the diagnosis of atrial cardiopathy and to determine whether atrial cardiopathy can be a new therapeutic target for DOAC.

## Author Contributions

YK performed the literature review and drafted the manuscript. ST performed critical revision of the manuscript. All authors contributed to the article and approved the submitted version.

## Conflict of Interest

The authors declare that the research was conducted in the absence of any commercial or financial relationships that could be construed as a potential conflict of interest.

## Publisher's Note

All claims expressed in this article are solely those of the authors and do not necessarily represent those of their affiliated organizations, or those of the publisher, the editors and the reviewers. Any product that may be evaluated in this article, or claim that may be made by its manufacturer, is not guaranteed or endorsed by the publisher.
